# Semiochemical delivery systems based on natural polymers to attract sand flies (Diptera: Psychodidae)

**DOI:** 10.1186/s13071-023-05931-w

**Published:** 2023-08-29

**Authors:** Ana Carolina Bueno, Vicente Estevam Machado, Flávia Benini da Rocha Silva, Fernanda Isadora Boni, Beatriz Stringhetti Ferreira Cury, Maria Palmira Daflon Gremião, Mara Cristina Pinto

**Affiliations:** 1 Department of Biological Sciences, School of Pharmaceutical Sciences, São Paulo State University (UNESP), Rodovia Araraquara-Jaú, km 1, Araraquara, São Paulo 14800-903 Brazil; 2 Department of Drugs and Medicines, School of Pharmaceutical Sciences, São Paulo State University (UNESP), Rodovia Araraquara-Jaú, km 1, Araraquara, São Paulo 14800-903 Brazil

**Keywords:** Attractiveness, 1-hexanol, Kairomones, *Lutzomyia longipalpis*, Release systems

## Abstract

**Background:**

The successful use of semiochemicals to attract insects to traps is based on research on the most suitable compounds and their release profiles over time. Based on the group's promising results, matrices with a more adequate release profile and more eco-friendly properties for the release of 1-hexanol were developed. To use a more suitable prototype in the field, the most promising systems were added to a capsule and evaluated in a wind tunnel. Behavioral experiments were performed using the sand fly species, *Lutzomyia longipalpis*, to evaluate the efficacy of the proposed system.

**Methods:**

Different delivery systems were developed by varying the polymer (gellan gum and pectin) ratio, crosslinker (aluminum chloride) concentration, and glutaraldehyde removal.The delivery systems were loaded with 1-hexanol, and their release profiles were evaluated using gravimetric analysis under ambient and high-humidity conditions. When the matrix system was placed inside a plastic container, modulations in the active release profile were observed and the system could be reused. Actid attraction behaviors of the sand fly species, *Lu. longipalpis*, were evaluated in a wind tunnel when exposed to 1-hexanol-loaded release systems at different times.

**Results:**

Among the four formulations evaluated, System 2 (gellan gum and pectin in a 1:1 ratio with 5% aluminum chloride) exhibited the most promising release profile, with greater uniformity and longer compound release time. The maximum 1-hexanol release uniformity was achieved over a longer time, mainly every 24 h, under both ambient and high-humidity conditions. System 2 can be reused at least once with the same structure. The wind tunnel trials exhibited efficient activation and attraction of *Lu. longipalpis* to 1-hexanol after 24, 48, and 72 h in System 2 placed inside the capsules.

**Conclusions:**

The polymeric matrix supplemented with 1-hexanol and introduced in plastic capsules showed promising results in attracting sand flies. This system can be used as a solution for other attractive compounds as well as in other applications where their release needs to be controlled or prolonged.

**Graphical Abstract:**

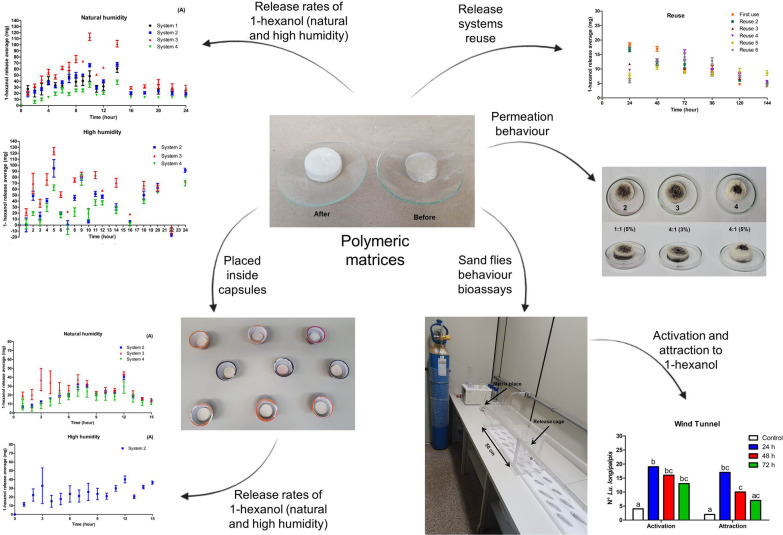

## Introduction

Sand flies (Diptera: Psychodidae) are hematophagous, holometabolous insects [1]. They are natural vectors of viruses, bacteria, and protozoan *Leishmania* [2]. Hematophagous insects exhibit an olfactory response to chemical compounds for obtaining blood meal sources [3].

Among the compounds characterized in the allelochemical category, kairomones are volatile compounds produced by organisms of one species that benefit individuals of another species [4]. Some kairomones produced by vertebrates (e.g., carbon dioxide, lactic acid, ammonia, and 1-octen-3-ol) have been evaluated in different groups of insects, including culicids, triatomines, keratopogonids, and simulids [5–9].

Previous studies have investigated the attractiveness of kairomones, such as octenol and 1-hexanol, to sand flies in *Nyssomyia neivai* species [10, 11]. For *Lutzomyia longipalpis*, octenol and nonanol exhibited better attractiveness responses in females and heptanol responses in males [12]. The evaluated alcohols are present in human sweat and produced by bacteria [13]. In all the cited studies, the alcohols were placed in capillary release systems on a string or filter paper posed as traps.

An important aspect of chemical ecology studies is the release of compounds into the environment to attract insects and improve insect capture. To ensure the success of these capture strategies, evaluating both the appropriate attractive compounds and best way to release them into nature is necessary.

Relevant advances have been made regarding systems for releasing compounds that attract insects of agricultural or epidemiological importance [14–17]; however, some challenges have been identified in the development of these systems, mainly owing to their use in unpredictable environments with large variations in temperature and humidity. Thus, the development of new systems that allow the modulation of the release rates of attractive compounds at pre-established time intervals and constant ratios attempts to overcome these challenges.

Gellan gum and pectin are natural hydrophilic polymers, commonly used in the pharmaceutical industry as drug delivery system matrices. Upon contact with water or humidity, these polymers swell, forming gels that act as barriers to control the release rate of drugs [18]. Despite differences in the release environment, drugs, and insect-attractive compounds, the ability to control the release of active compounds associated with the demand for natural products has prompted research on the possible use of these natural polysaccharides in the development of devices to attract insects. In a previous study, a polymer matrix system based on gellan gum, pectin, and crosslinkers glutaraldehyde and aluminum chloride was used as a potential device to modulate the release of 1-hexanol, which was reported to attract the sandfly *Ny. neivai* (patent number: BR 10 2019 001965 4). However, certain aspects of the first prototype must be improved. For instance, disposal of glutaraldehyde, used in this formulation, poses an environmental problem. In addition, the system maintained the release of 1-hexanol at attractive levels for sand flies for only 24 h in wind tunnel experiments [19].

In this context, the present study was aimed at modifying the chemical structure of this precursor device to make it more environmentally acceptable by either removing glutaraldehyde or evaluating the possibility of reusing the material matrix. Furthermore, stabilizing the release of the compound for a longer time is important to prolong the attractiveness of sand flies.

## Materials and methods

### Polymer matrix release system development

Following a previously reported approach, under patent number BR 10 2019 001965 4 [16], with some modifications, four delivery systems were developed using gellan gum (CG-LA, 97%, CP Kelco) and pectin (LM-5206 CS, 99%, CP Kelco) blends at different mass ratios. Different concentrations of aluminum chloride were used as a crosslinker. The system formulations were as follows:

- System 1: Gellan gum and pectin (1:1 ratio) with 3% aluminum chloride.
- System 2: Gellan gum and pectin (1:1 ratio) with 5% aluminum chloride.
- System 3: Gellan gum and pectin (4:1 ratio) with 3% aluminum chloride.
- System 4: Gellan gum and pectin (4:1 ratio) with 5% aluminum chloride.

In the first attempt to develop these systems, aqueous dispersions of the polymeric blends were prepared, and ionic cross-linking with aluminum chloride was subsequently conducted for 30 min, with the solution being added to the polymeric dispersions under magnetic stirring. Thereafter, the cross-linked gel was centrifuged at 3000 rpm for 10 min and stored at − 20 °C, followed by freeze-drying for 72 h [16]. However, to minimize the differences in the physical structures of the four release systems, the development approach was modified. After centrifugation, 30–60 min of storage in a common refrigerator (– 6 °C) was conducted, followed by conditioning at − 20 °C, and the freeze-drying time was reduced to 40 h.

### Evaluation of the release rates of 1-hexanol in the polymeric systems

To evaluate the release rate of 1-hexanol (Sigma-Aldrich, 98%), 2 ml of the compound was carefully injected into the middle of the system using a syringe and needle. Subsequently, the systems were weighed on a properly calibrated analytical balance every 1 h for 24 h and every 24 h for 240 h under ambient humidity (53%; 28 °C) and every 1 h for 24 h under high humidity (81%; 25 °C). The release rate was defined as the difference between the final and initial weights of the polymer system. Three replicates were used for each assay.

### Evaluation of the release rates of 1-hexanol in polymeric systems placed inside capsules

Since delivery systems are to be used in a field where temperature and humidity fluctuations are significant, the matrices were also evaluated under high humidity conditions.

**Fig. 1:**
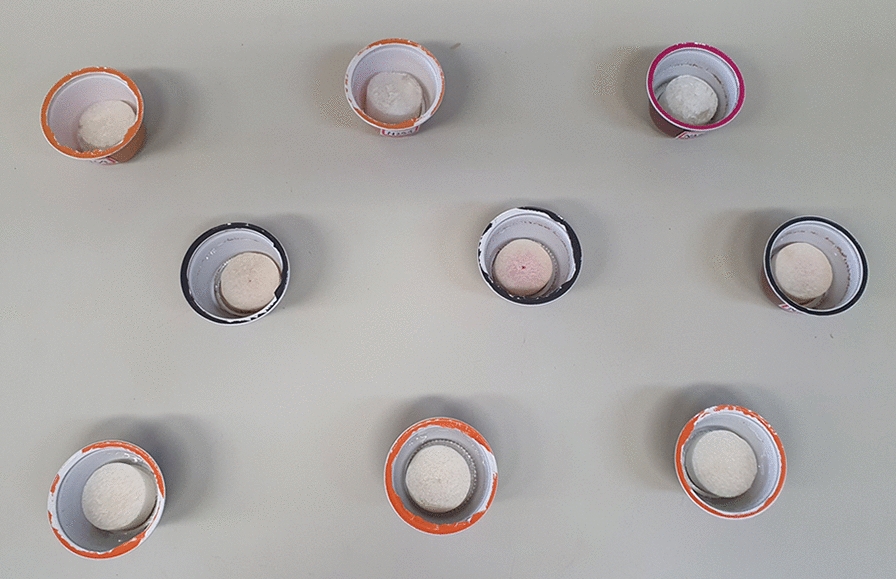
Polymeric matrices of different formulations placed inside small plastic containers for 1-hexanol controlled release tests

After the first experiment, Systems 2, 3, and 4 were placed inside small plastic containers with open upper openings. These containers were commercial coffee capsules measuring 4 × 5 cm (w × h) with an aluminum base (Fig. 1). They were weighed every 1 h for 15 h and every 24 h for 288 h under ambient humidity (47%; 27 °C) and high humidity (80%; 28 °C). Three replicates were used for each assay.

### Permeation behavior

To observe the permeation behavior of 1-hexanol in the matrix visually, Bordeaux red dye (0.5 g) was mixed with 2 ml of the compound, which was inserted into the system using a syringe and needle.

### Reuse assessment

Based on the results of the previous experiments, System 2 was selected to evaluate the possibility of reuse.

The matrices (*n* = 3) were placed inside commercial coffee capsules until all the 1-hexanol was released for up to six consecutive trials. The systems were weighed every 24 h for 144 h under ambient humidity (55%; 27 °C).

### Sand fly behavior bioassays

Behavioral bioassays were performed in a wind tunnel at controlled temperature (22 ± 1 °C) and humidity (65 ± 1%) [10].

Thirty *Lu. longipalpis* sandfly females, 7–10 days old, were used in the trials. The insects were maintained in colonies at the Parasitology Laboratory of the School of Pharmaceutical Science, São Paulo State University [20]. For the trials, the insects were placed in groups of three in ten release cages (*n* = 30/trial).

**Fig. 2:**
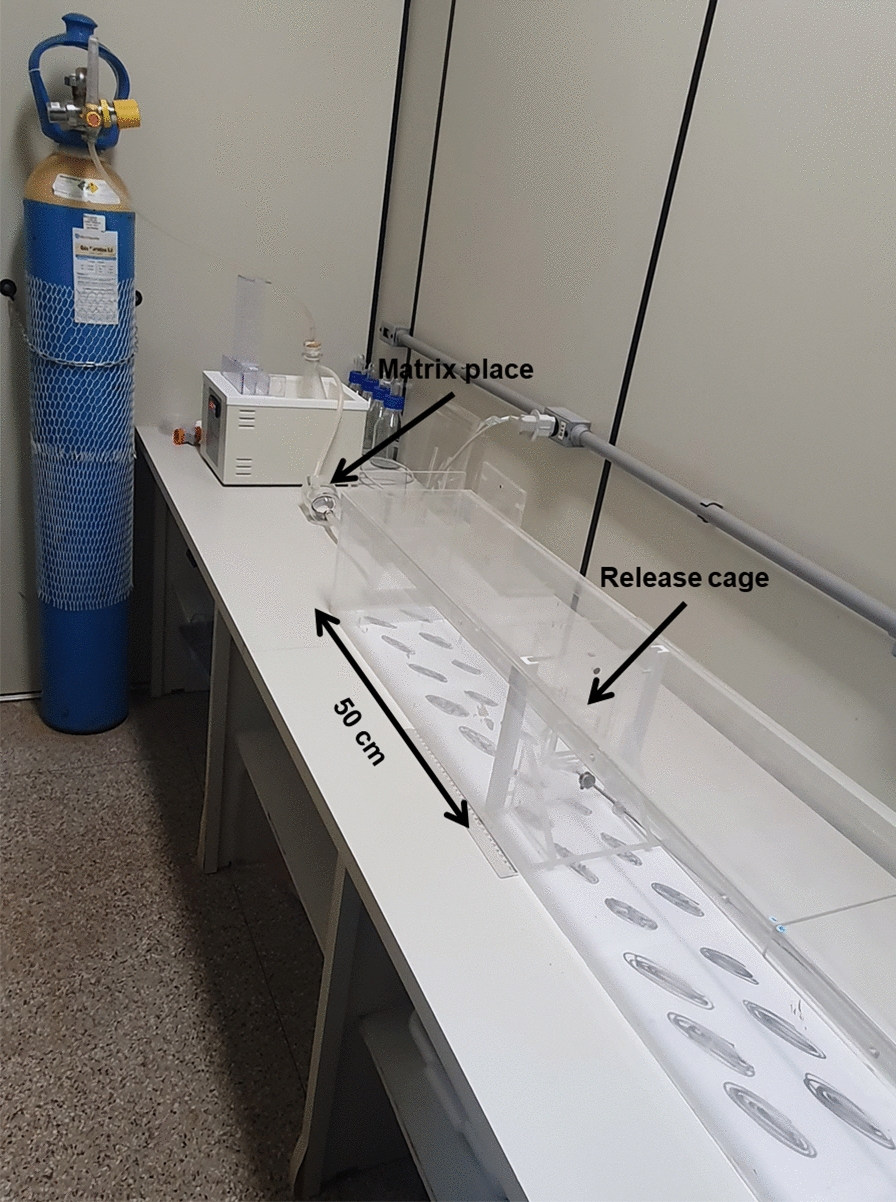
Wind tunnel. Sand flies in release cage placed 50 cm from the matrix

The wind tunnel was prepared and sanitized, and the insects were placed 50 cm away from the stimulus with a constant airflow of 1 l/min (Fig. 2).

All trials lasted for 2 min, and both activation (insects that left the release cage) and attraction (insects that reached the stimulus) were evaluated using release System 2 placed in capsules with and without 1-hexanol (control).

Experiments were performed using unfed *Lu. longipalpis* females at 24, 48, and 72 h after the addition of 1-hexanol to the release system.

A Chi-square test was used for the statistical analysis of the differences in the proportions of sand flies that were activated and attracted by release System 2 with 1-hexanol and control at each time point. BioEstat software (version 5.0) was used for the meta-analysis, with 95% significance (*P* < 0.05).

## Results

### Physical structure of the release systems

**Fig. 3:**
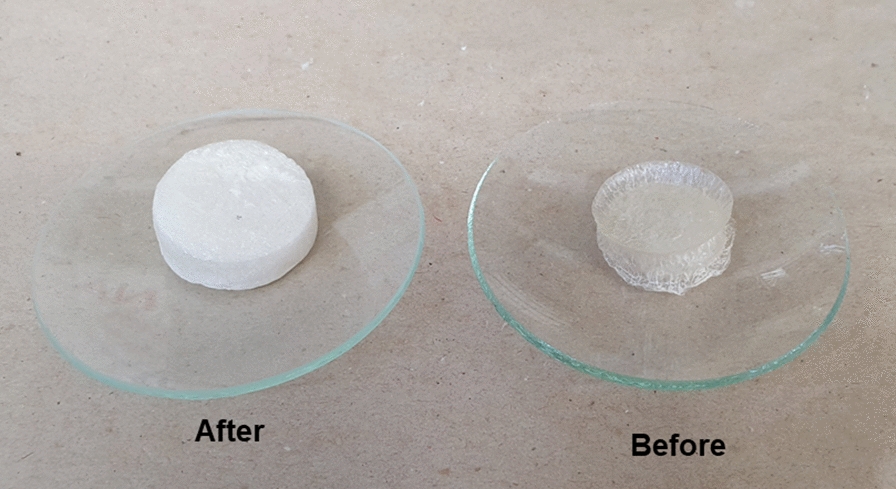
Physical differences between polymeric matrices before and after standardization of the matrix development protocol

The initial protocol for system manufacturing was changed, as described ("Polymer matrix release systems development" section). The matrices were observed during the drying process, according to a previous study; however, irregular drying and structural deformation were observed (Fig. 3). The sample freezing and drying were then altered. The matrices were maintained at a temperature of 4 °C before − 20 °C and had a shorter lyophilization time (40 h instead 72 h).

### Evaluation of 1-hexanol release rates in the release systems

**Fig. 4:**
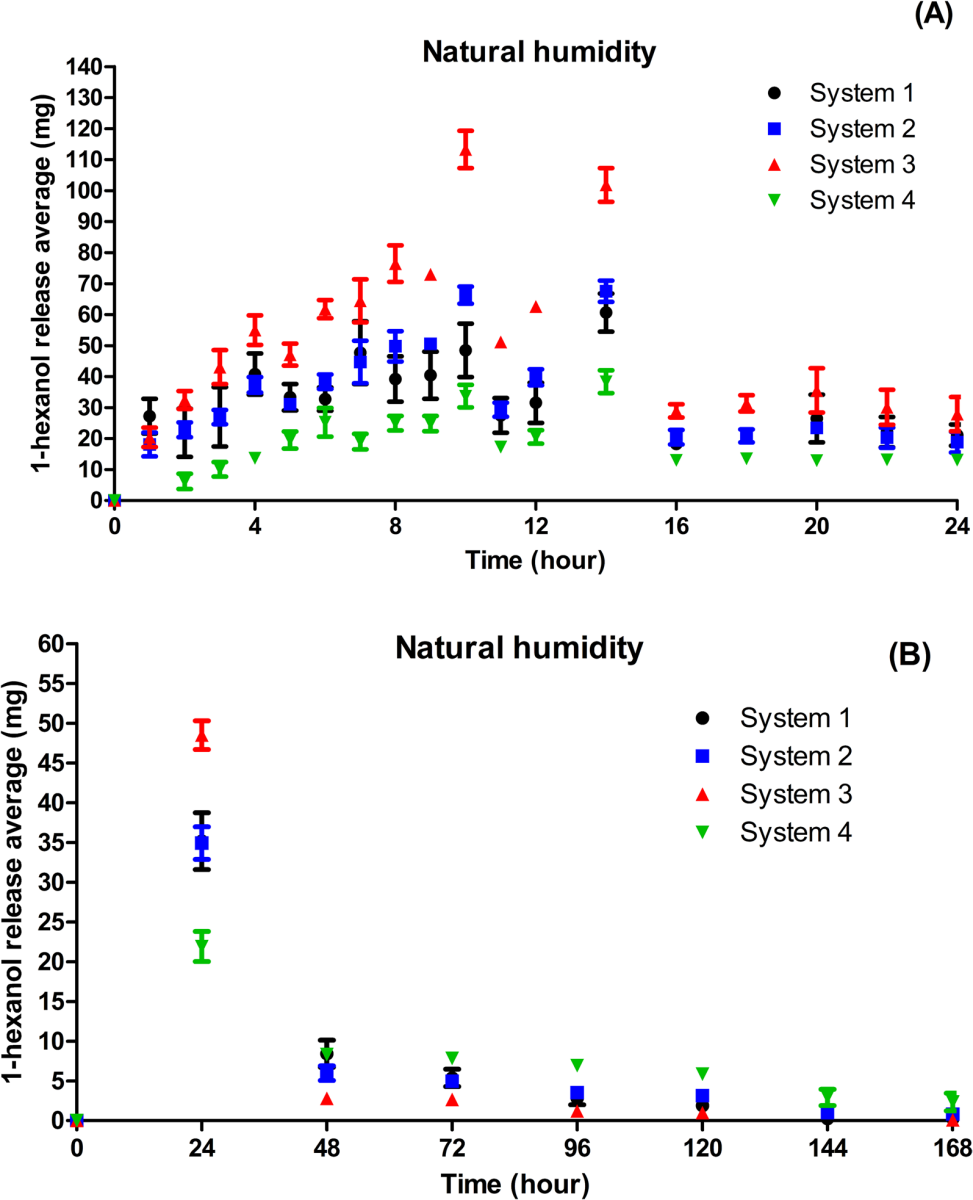
Ambient humidity. **A** Average 1-hexanol released (mg) in Systems 1–4 in the first 24 h. **B** Average 1-hexanol released in Systems 1–4 every 24 h

After manufacturing the matrices, the release profiles of the compounds were evaluated under ambient and high-humidity conditions. Under ambient humidity, 1-hexanol was completely volatilized between days 6 and 7 (Fig. 4). System 3 released the highest amount of 1-hexanol during the first 24 h, whereas System 4 released the lowest. From the 16th hour, the release of 1-hexanol stabilized.

System 1 was not used in subsequent trials since it exhibited a release rate close to that of System 2 with a high deviation among the replicates.

**Fig. 5:**
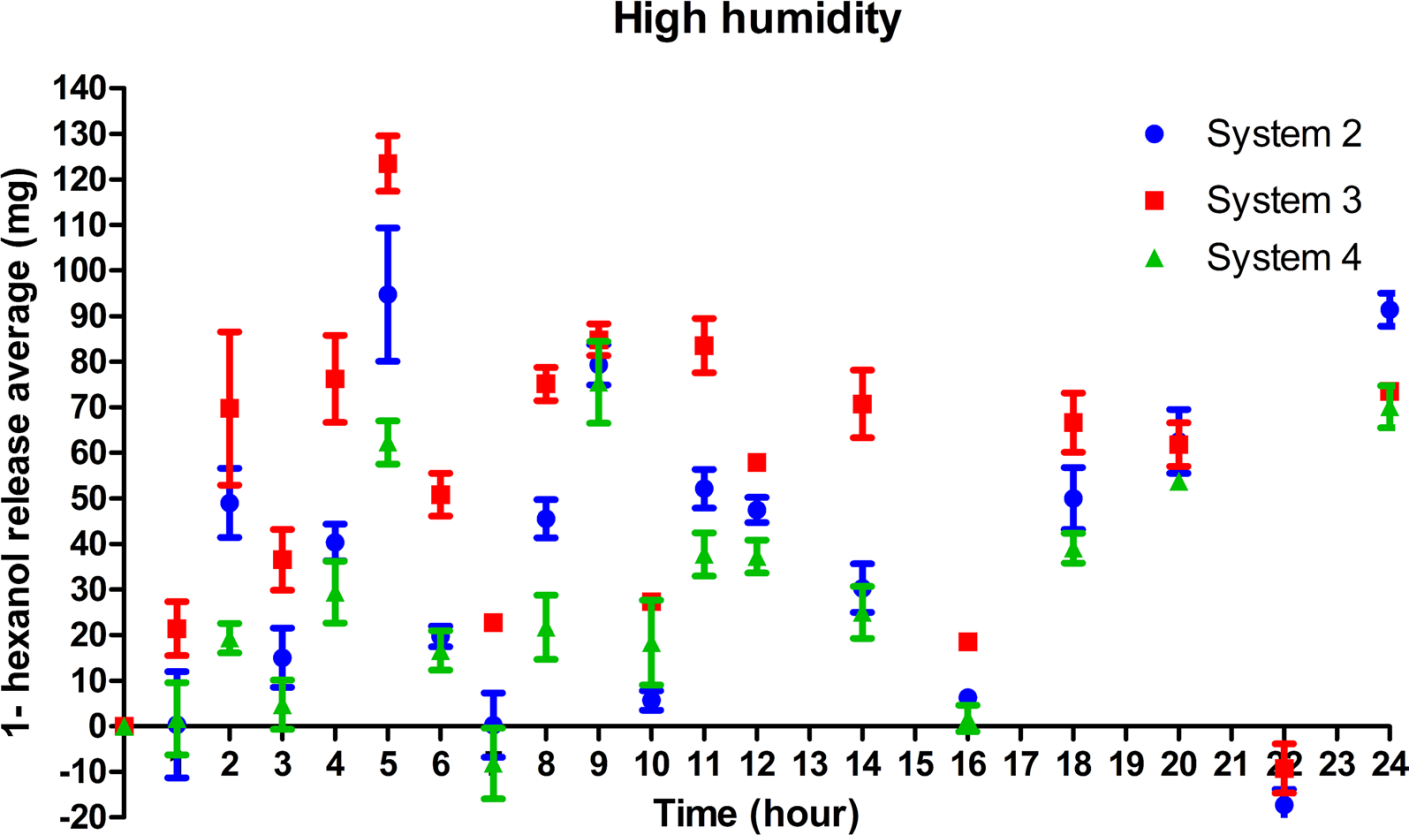
High humidity. Average 1-hexanol release (mg) in Systems 2–4 during the first 24 h

The evaluation of the 1-hexanol release profile at high humidity indicated that at the 22nd hour the compound was completely volatilized in all release systems. Thereafter, the systems absorbed water from the environment with an increase in weight after 24 h (Fig. 5). Despite the highest oscillation in the release rates compared with the previous test, the pattern was similar, with Systems 3 and 4 exhibiting the highest and lowest release rates, respectively.

### Evaluation of 1-hexanol release rates in the release systems placed inside capsules

**Fig. 6:**
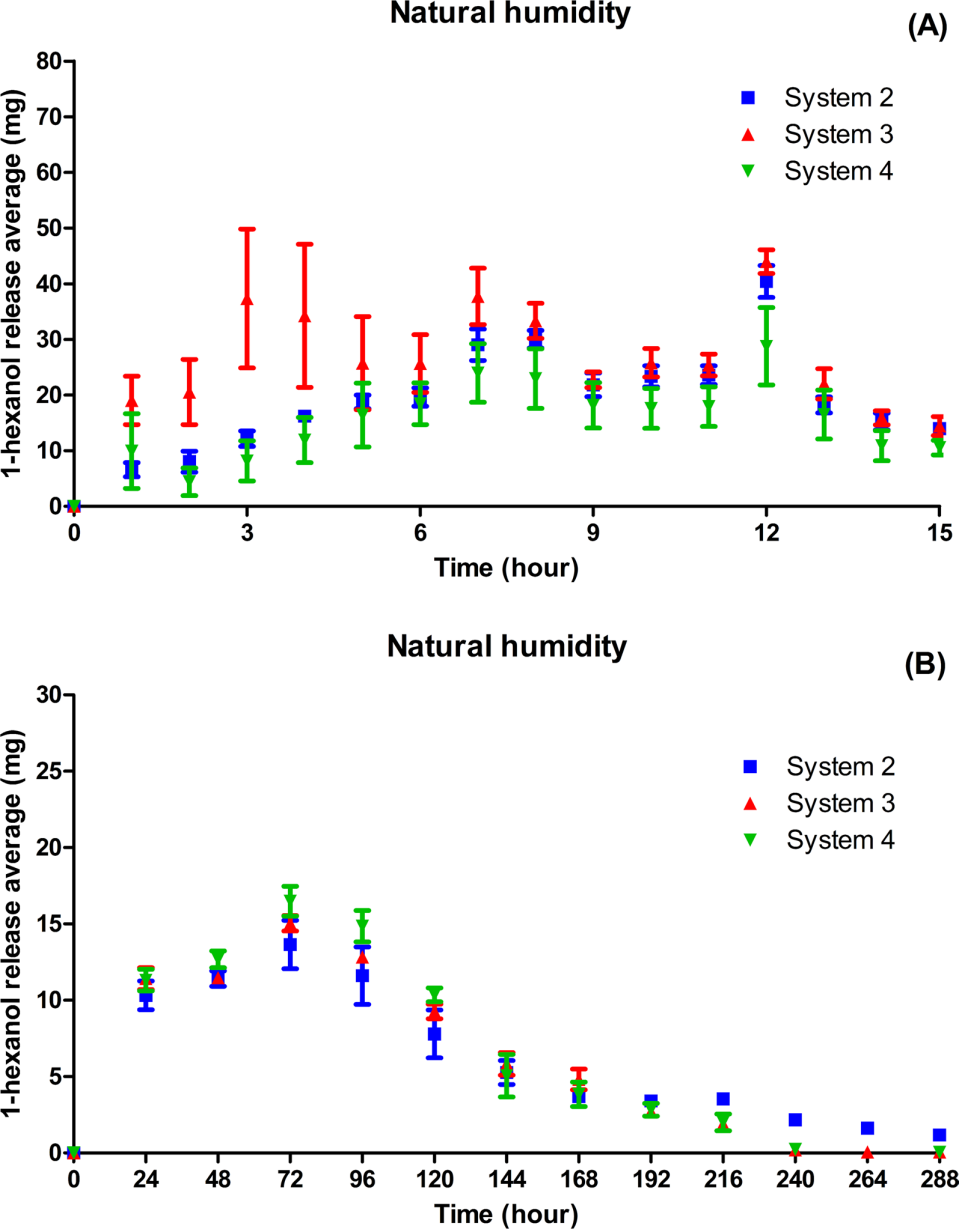
Ambient humidity. Average 1-hexanol release (mg) in Systems 2–4 placed inside capsules in the first 15 h (**A**). Average 1-hexanol release (mg) in Systems 2–4 placed inside capsules every 24 h (**B**)

The matrices were packed into commercial coffee capsules to find a way to take the matrices into the field, make the volatilization more stable and uniform, and achieve greater control over the compound release rate. Under these conditions, the three systems exhibited a pattern of 1-hexanol release (Fig. 6).

Systems 2 and 4 exhibited similar release profiles with peaks at the 7th, 8th, and 12th hour.

System 2 exhibited greater uniformity with the lowest standard deviation in the volatilization of the compound among the replicates. In addition, the compound lasted longer in System 2, up to 288 h, whereas in Systems 3 and 4, it lasted up to 240 h. Therefore, System 2 was selected for subsequent trials.

**Fig. 7:**
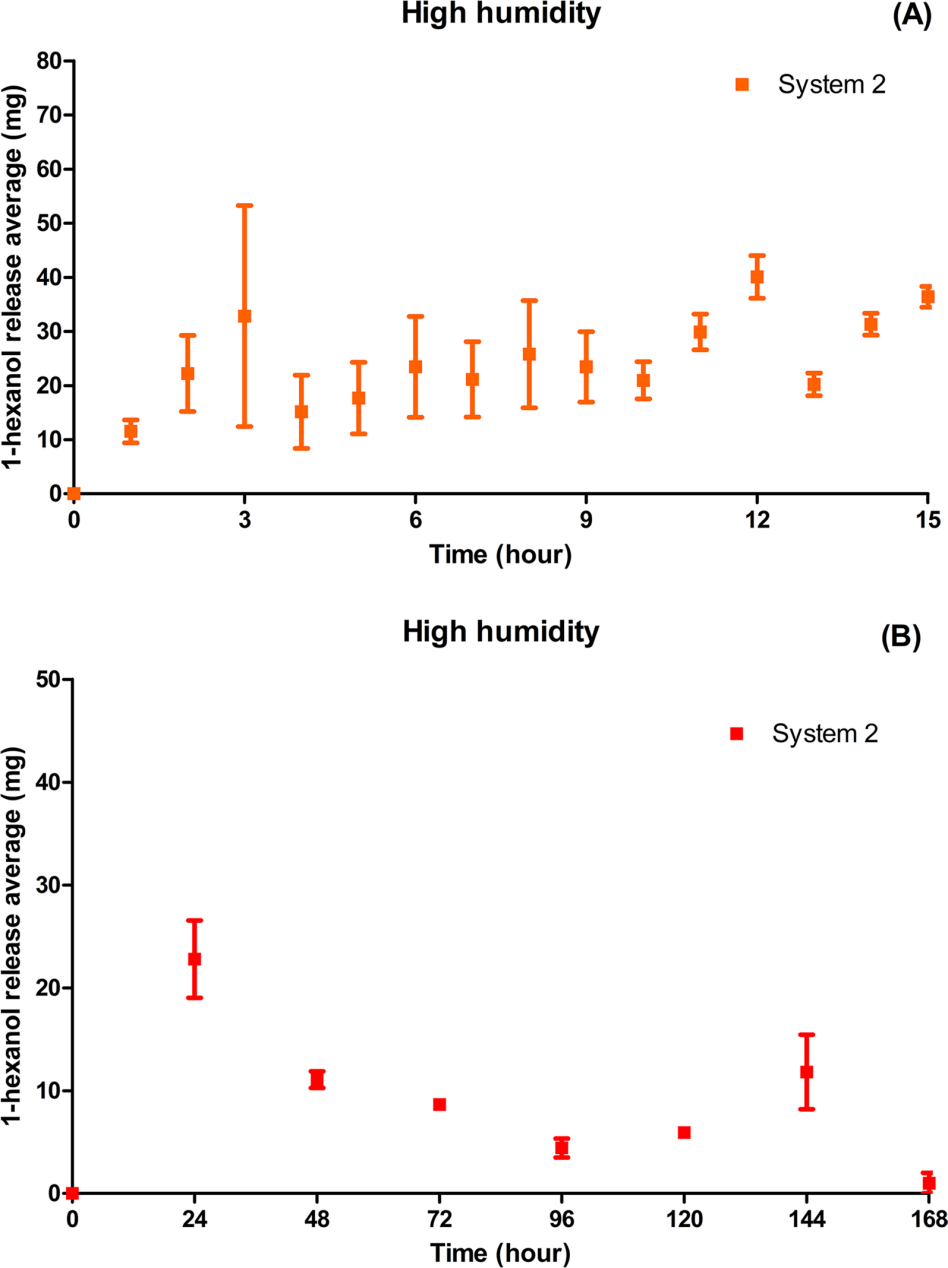
High humidity. **A** Average 1-hexanol release (mg) in System 2 placed inside capsules in the first 15 h. **B** Average 1-hexanol release (mg) in System 2 placed inside capsules every 24 h

At high humidity, System 2 inside the small containers exhibited significant uniformity in the release rate of 1-hexanol compared to the experiment without capsules (Fig. 7). On the 7th day (168 h), the system released the entire volume of the compound and began to absorb water.

### Permeation behavior

**Fig. 8:**
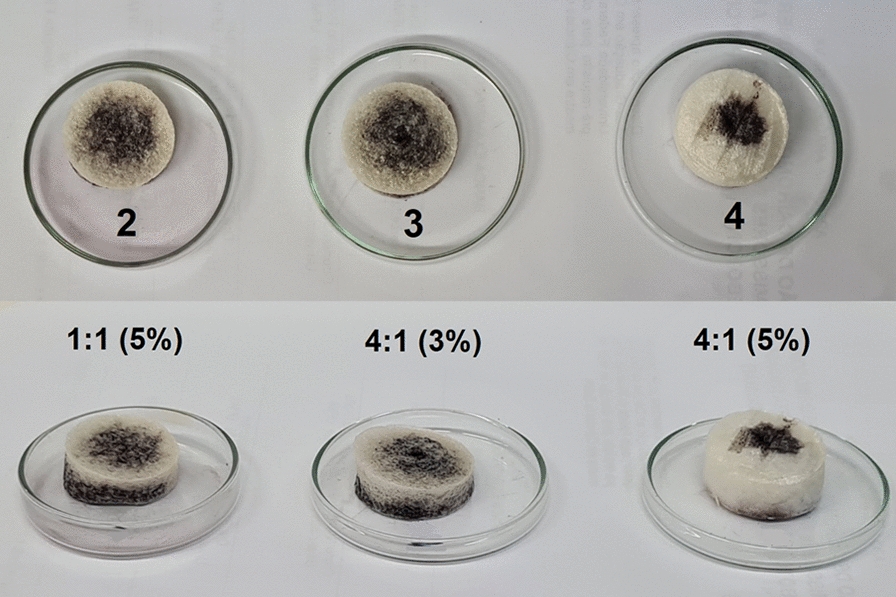
Permeation of 1-hexanol with Bordeaux red dye in Systems 2, 3, and 4 in top and lateral view

To understand the observed release behavior, the distribution of 1-hexanol across the matrices was evaluated. A similar compound distribution was observed in Systems 2 and 3; in both matrices, the compound was evenly dispersed at the bottom. In System 4, the compound was more concentrated and less distributed (Fig. 8).

### Release systems reuse

**Fig. 9:**
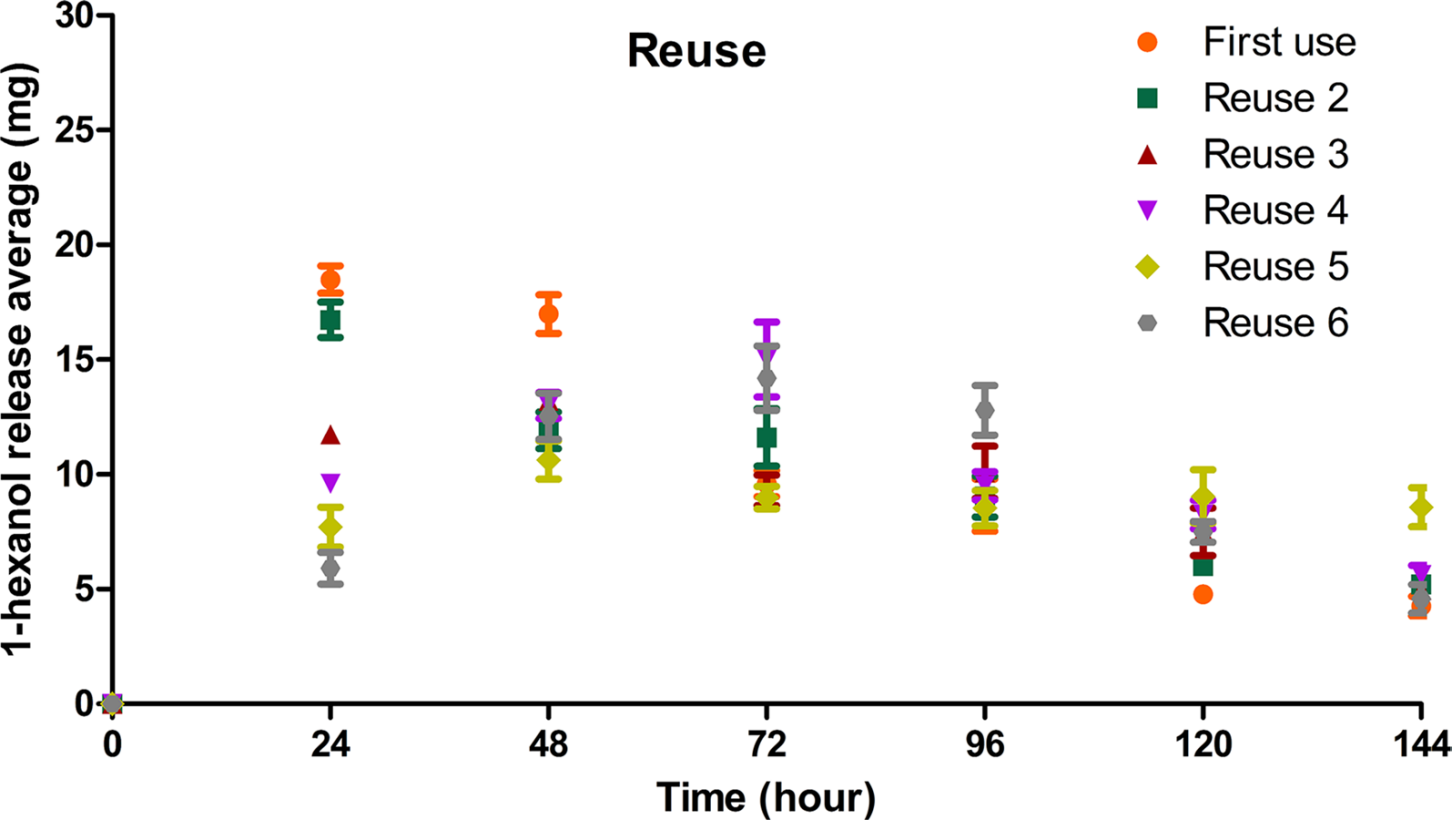
Repetition of the average 1-hexanol release rate in System 2 placed inside capsules in ambient humidity every 24 h up to 6 times

The experiment evaluating the reuse capacity of the matrices after all the 1-hexanol was released exhibited stability in the release rates with each reuse of the material (Fig. 9). The first use of release System 2 and the second use showed the highest release rates during the first 24 h, with an average release of 17 mg/h. The other reuses exhibited uniform volatilization according to reuse, with an average release of 10 mg/h at 96 h, which dropped to 5 mg/h at 144 h.

### Sand fly behavior bioassays

In wind tunnel experiments, the behavior of *Lu. longipalpis* females to 1-hexanol released from System 2 inside the capsules was evaluated 72 h after the introduction of the compound into the matrices.

**Fig. 10:**
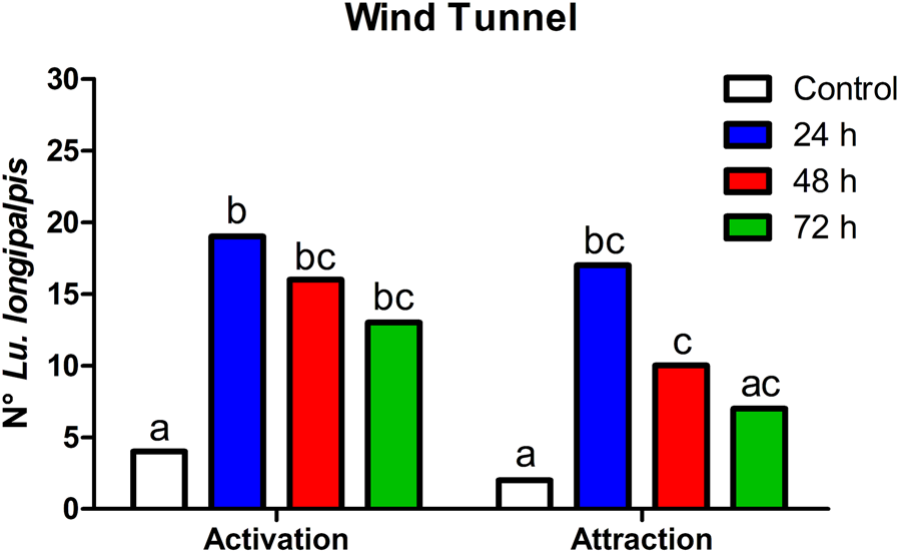
Activation and attraction behavior of *Lu. longipalpis* females in the wind tunnel with 1-hexanol in System 2 placed inside capsules. For each behavior of activation or attraction, the bars with different letters are significantly different (*n* = 30/trial)

A statistically significant difference was observed in the activation behavior of *Lu. longipalpis* females between 1-hexanol and the control for up to 72 h. For attraction, differences were observed for up to 48 h (Fig. 10). In the control group, four insects were activated and two insects were attracted. After 24 h of 1-hexanol introduction, 19 insects were activated (*χ*^2^: 15.86, *df*: 1, *P* < 0.0001) and 17 were attracted (*χ*^2^: 17.32, *df*: 1, *P* < 0.0001) with a 26.57 mg/h 1-hexanol release rate. After 48 h, 16 insects were activated (*χ*^2^: 10.80, *df*: 1, *P* = 0.0010) and 10 insects were attracted (*χ*^2^: 6.66, *df*: 1, *P* = 0.0098) with a 16.17 mg/h 1-hexanol release rate. After 72 h, the sand flies exhibited significant behavioral responses only during activation; 13 insects were activated (*χ*^2^: 6.6484, *df*: 1, *P* = 0.0099) and 7 were attracted (*χ*^2^: 3.2680, *df*: 1, *P* = 0.0706) with a 22.67 mg/h 1-hexanol release rate.

## Discussion

Light traps are the main strategy used to collect sand flies in a field [21]. In addition, the association of light with attractive compounds may enhance sandfly capture [22]. Insect chemical ecology studies have evolved the search for effective attractive compounds and the best way to release them into the environment.

The development of drug delivery systems is an area of research that has advanced significantly [23]. Some initiatives have been made to apply this knowledge to insect chemical ecology to find better ways of releasing attractive compounds and improving insect traps. For instance, a new system using clay, amylose, and zeolite was developed to release aggregation pheromones and control one of the main pests of the coconut palm, *Rhynchophorus palmarum * [15].

Some authors have reported that the use of polymeric matrices to release attractive compounds for *Aedes aegypti* increased the stability and durability of the attractive compounds, achieving efficient attraction and capture of insects [14].

Gellan gum and pectin form hydrogels, which are three-dimensional cross-linked networks formed by polymer chains with the potential to absorb water or organic compounds and preserve the material structure [24]. In a previous study, these materials were used in the development of matrices to release 1-hexanol and attract sand flies [16]. The aim of this study was to improve the matrices by removing glutaraldehyde and more effectively attract sand flies by releasing 1-hexanol for more than 24 h, as observed by Machado et al. [19].

These results demonstrated that the withdrawal of glutaraldehyde did not render the matrices unfeasible. In contrast, the results indicate uniformity and a pattern in the release of 1-hexanol. In addition, the total volatilization of 1-hexanol indicated total shedding of the molecules, conferring quality to the material.

The formation of a homogeneous polymeric dispersion is mandatory, which when dried efficiently creates a dense mesh and retains the incorporated active material with pores of a similar structure. This material allows the subsequent controlled diffusion of the active material into the environment. Thus, the optimization and standardization of the manufacturing process are important steps as they affect the design of the system.

In the first manufacturing process of the matrices, physical differences, such as dryness, and consequently, great instability in the release rates of attractive compounds, were observed. After optimizing the manufacturing process, obtaining physical standardization, which contributes to the controlled release of the attractant (as described in "Physical structure of the release systems" section), was possible.

Tests were conducted under high humidity to simulate field conditions and a possible environment for sandfly capture. Sand flies develop primarily in wild environments and generally in vegetated and shaded areas [25, 26].

The strategy of placing the release systems inside the capsules demonstrated a greater stability of 1-hexanol release over a longer time. This event was probably caused by the reduced contact surface, in which the detachment of the 1-hexanol molecules was directed along a single vertical route.

Systems 2, 3, and 4 placed inside the capsules exhibited similar 1-hexanol release profiles. However, during the first 15 h, System 3 exhibited a larger standard deviation in the release of 1-hexanol. This indicates that Systems 2 and 4 exhibited greater stability in the 1-hexanol release profile than System 3, probably owing to the use of 5% aluminum, which was more efficient in matrix crosslinking, forming a more uniform structure with a smaller number of defects. In System 3, the 3% concentration of aluminum chloride promoted loosening of the matrix structure in relation to that of System 2 at 5%, which contributed to the faster migration of 1-hexanol molecules. System 4 exhibited a lower release rate, indicating greater entrapment of the molecules owing to its dense structure due to crosslinking at 5% and the presence of a higher ratio of gellan gum. Presumably, this system, in contact with humid air, resulted in the formation of a dense gel layer that acted as a barrier to compound diffusion. Thus, the network formed by crosslinking at 5%, and the highest amount of gellan gum facilitated the achievement of a more stable and long-lasting release rate.

These observations were supported by visual permeation analysis of 1-hexanol (Fig. 8). This effect, especially in System 4, probably occurred owing to the higher concentration of gellan gum and the crosslinking degree with the higher concentration of aluminum, resulting in the formation of a denser, more organized matrix with smaller pores, which held the compound and prevented migration after application.

Among the evaluated systems, System 2 was chosen owing to its greater uniformity and longer retention time.

The reuse of materials contributes to waste reduction and sustainability, and a reuse test for System 2 was conducted to evaluate this aspect. The results demonstrated that the second reuse presented a pattern of 1-hexanol release similar to that of the first use. During the first 24 h, both tests exhibited the highest 1-hexanol release average; however, subsequently, this release average decreased according to the number of applications. At 48 h, the release rate slightly increased from the third to the sixth reuse. Considering the hydrophobic and biodegradable nature of the matrices, this may indicate the degradation of the polymeric matrix or disorganization of its structure after the third use.

Wind tunnel behavioral trials showed that System 2 had a positive response to attraction for *Lu. longipalpis* females for up to 48 h after 1-hexanol was introduced into the system. Although 72 h was still an attractive response for females, this result was not statistically different from that of the control experiment. These results were better than those reported in a previous study on 1-hexanol for *Ny. neivai*, which was effective only for up to 24 h [19]. Further investigations are necessary to evaluate the efficiency of release systems for collecting sand flies in the field.

## Conclusions

Our findings demonstrated that the system based on gellan gum and pectin supplemented with 1-hexanol and introduced in plastic capsules showed promising results in attracting the sand fly *Lu. longipalpis*. It was possible to modulate the release of the compound for a longer time and prolong the attractiveness of sand flies. In addition, these devices use matrices composed of environmentally friendly materials and their reuse will reduce the impact on the environment. This system can be used as a solution for other attractive compounds as well as in other applications where their release needs to be controlled or prolonged.

## Data Availability

All relevant data are within the paper.
